# Improving Chinese patients’ autonomy in medical decision-making through policy frameworks

**DOI:** 10.3389/fpubh.2025.1553721

**Published:** 2025-06-18

**Authors:** Gaoyuan Zhai

**Affiliations:** Law School, Tianjin University, Tianjin, China

**Keywords:** China, medical decision-making, patient autonomy, medical familism, policy framework

## Abstract

As rational organisms, human beings not only require a dignified existence in social relationships, but also require the ability to create themselves according to their own will and determine the development trajectory of their own lives. Medical decisions involve the patient’s life, health, body and other factors that are most closely related to the person. Individuals with dignity should make their own medical decisions and decide according to their own wishes. In Chinese society, due to the profound influence of Confucianism, medical familism occupies an important position in the doctor-patient relationship. Under the medical familism model, medical decisions are not the patient’s own private issues, but important matters that affect the future development of the entire family. When family members offer advice on medical decisions, in a positive sense they contribute overall wisdom to the patient’s medical treatment; however, in a negative sense, this may be seen as compressing the patient’s autonomy. In contemporary Chinese society, medical disputes occur frequently, and the harmonious doctor-patient relationship appears cracked. Improper exercise of medical decision-making is an important cause of many medical disputes. This article adopted the research methods of case analysis and comparative analysis. By analyzing the shortcomings of China’s current policy framework on medical decision-making and combining the influence of traditional Confucianism on China’s medical decision-making model, it proposed methods to improve China’s policy framework in order to enhance patients’ autonomy in medical decision-making.

## 1 Introduction

Everyone has the freedom to determine the development of his or her own personality according to his or her own wishes. Medical autonomy reflects the patient’s self-determination of his or her own medical activities and emphasizes respect for free will. Due to unclear provisions on medical autonomy in Chinese law, patients’ medical wishes are not respected or even ignored, resulting in patients being unable to decide on medical activities according to their own wishes. On August 31, 2017, a pregnant woman M in Yulin City, Shaanxi Province, was in great pain due to difficult labor. She repeatedly asked her family for a caesarean section. Medical staff also made this suggestion to her family, but the patient’s family refused to agree. M jumped off the building due to unbearable pain and eventually died (1). M strongly requested a cesarean section many times. At that time, she was conscious, able to judge the risks of a cesarean section, and had the capacity to make medical decision to have a cesarean section. However, her medical wishes were ignored. The deprivation of medical decision-making indirectly led to M’s death.

In Chinese society, although the importance of family in medical decision-making is emphasized, we must acknowledge that the patient’s close relatives or guardians do not always make medical decisions with the patient’s best interests in mind. Many patients do not have the capacity to make medical decisions due to age, injuries, mental conditions, etc. At this time, their autonomy rights and interests are often not respected as they should be, and incidents that infringe on the patients’ lives, health, and physical bodies occur frequently. For example, in the “welfare home girl’s uterus removal case” that occurred in Nantong City, Jiangsu Province in March 2005, two mentally disabled girls were instructed by the head of the social welfare home to have their uteruses removed by doctors, simply because the two girls’ menstruation increased the difficulty of care during their menstrual period (2). As a result, the two mentally disabled girls had their uteruses removed and permanently lost their right to reproduce. Tom L. Beauchamp and James F. Childress proposed four principles of biomedical ethics: respect for autonomy; nonmaleficence; beneficence; justice (3). These four basic principles are based on the moral requirements of the general public, represent the common cognition and tradition of the general public, and are the methods and standards for testing most cases in the field of life and medical ethics. In the above case, the two girls were mentally disabled patients who did not have the corresponding medical decision-making ability. As their guardian, the welfare home should protect the best interests of the patients in medical decision-making matters. However, the person in charge of the welfare home reduced the difficulty of care by depriving the patients of their normal physiological functions. The medical methods and medical purposes did not meet the requirements of the proportionality principle, and even violated the principle of the best interests of the child in the Convention on the Rights of the Child. For the hospital that performed the hysterectomy, its behavior seriously violated the basic principles of nonmaleficence and beneficence.

In the “X refusal to sign case” that caused widespread discussion in Chinese society in 2007, L was sent to the hospital by her cohabiting boyfriend X due to life-threatening complications from difficult labor and cold. The hospital told X several times that L’s death would be inevitable if a caesarean section was not performed immediately for L. However, X always refused the doctor’s caesarean section for L, and even signed the consent form, “I refuse the doctor to perform a caesarean section on L, and the consequences are at my own risk” (4). As the hospital was unable to obtain X’s consent and did not dare to perform a cesarean section, which had a higher medical risk, it could only request a response from the hospital director, the police, and the Beijing Health Bureau to consent to the operation, but no organization dared to take responsibility. In desperation, the medical staff could only carry out rescue operations according to conventional methods. In the end, both L and the fetus in her belly died. In emergency clinical treatment situations, it often happens that the patient’s family members cannot reach a consensus on medical decisions. The tug-of-war among family members over medical decisions will undoubtedly delay the patient’s best treatment time. In this case, how to ensure that family members participate in expressing their opinions and avoid meaningless shirking of responsibility? After the patient’s family has finally reached a consensus on a medical decision after lengthy discussions and weighing of interests, how can we ensure that the patient’s family makes a medical decision that is in the best interests of the patient? These are important issues that need to be urgently addressed in Chinese law.

Differences in cultural traditions have led to the differentiation of medical decision-making paradigms in Chinese and Western societies, and Confucianism has shaped the uniqueness of China’s social structure (5). For Chinese people, the patient’s medical decision is an important matter for the entire family. On the basis of patient autonomy, more emphasis is placed on the participation of the family’s overall wisdom, which is significantly different from the patient autonomy theory in Western countries. On May 28, 2020, the Civil Code of the People’s Republic of China was successfully passed. The Civil Code was called the “encyclopedia of social life” by Chinese scholars. However, the Civil Code’s provisions on patient medical decision-making are not perfect, and many issues related to patient autonomy are not fully clarified. In view of this, the article first discusses the formation and development of patient autonomy in the world, as well as the legislation on patient autonomy in other countries and regions. Then, combined with the Confucian cultural tradition of Chinese society, this article systematically analyzes the limitations of China’s social medical decision-making policy framework. Finally, on this basis, corresponding improvement suggestions are put forward, hoping to enhance patients’ autonomy in medical decision-making by improving the policy framework, thereby improving China’s level of human rights protection.

## 2 Informed consent and patient autonomy

As one of the three traditional academic majors, medical technology is mysterious and complex. As a layman in medicine, when patients needed help from doctors due to injuries or illnesses, he stepped into a strange land and was at a loss as to what to do (6). Rich medical knowledge enables doctors to have professional capabilities. Doctors have an in-depth understanding of human nature, pathology, and the mechanisms of the body, and know what kind of medical decisions are beneficial to patients. However, patients know nothing about complex medical knowledge, and their participation in medical activities is not a wise choice, which will hinder communication between doctors and patients. Therefore, in early medical activities, doctors do not talk too much with patients. Doctors usually do not explain much about the patient’s condition and the content, purpose, procedures, side effects, efficacy and other matters of the medical activities to be performed on the patient. As Hippocrates said: “Perform your duties calmly and skillfully, keeping the patient in the dark while you care for him. Give the necessary orders cheerfully and sincerely, distracting attention from what he is doing; sometimes rebuke, sometimes comfort and care, never revealing the patient’s future or present situation” (7).

### 2.1 The establishment and development of patient autonomy

During the Renaissance, the emerging bourgeoisie broke through the shackles of theology, rescued people from religious oppression, restored the value of human subjectivity, revived human personality, and sought freedom of thought and speech. During this period, medicine advanced in tandem with literature and art, and the doctor-patient relationship transitioned from a simple model of mutual trust to a medical contract model. Since the 1860s, the human rights movement in the West has promoted a change in the patient’s concept. Patients have sought to protect their own health rights, such as the right to terminate pregnancy and contraception, the rights of subjects in human experiments, and the right to access medical information. With the evolution of social civilization, the human rights movement has awakened patients’ awareness of their rights, and patients have increasingly emphasized their control over medical activities. This concept has quickly become a trend around the world. In Schloendorff v. Society of New York Hospitals in the United States in 1914, a doctor removed a fibroid tumor from an anesthetized patient during an examination. Since the patient had clearly requested not to undergo removal surgery before the examination, the patient sued the hospital to court (8). Judge Benjamin Cardozo of the New York State Court of Appeals stated in the ruling: “Every adult and of sound mind has the right to decide what to do with his or her own body; a surgeon who performs an operation without the patient’s consent constitutes a personal assault and should be liable for damages; Exceptions are made for operations performed in emergency situations where the patient is unconscious” (9). The case pioneered the use of the term self-determination, marking the first judicial recognition of a patient’s right to self-determination by a court ruling.

The principle of autonomy, as one of the four basic principles of medical ethics, aims to stipulate the subjective status of patients in medical activities, so that patients can make relevant medical decisions according to their own wishes without illegal interference from others (10). In Kant’s philosophical concept, autonomy means that a subject with free will is not dominated by another subject with free will (11). As rational beings, humans are able to judge the value of their actions and be responsible for the consequences of their actions. Hegel believed that “man’s highest mission is to become a person” and “the command of law is to become a person and respect others as human beings” (12). As long as any person’s actions involve only himself, he himself is the supreme sovereign, and his independence is absolute in rights (13). No one can force others to do something or not to do something, even if it is an action that is beneficial to themselves, unless it is necessary in an emergency or to protect the public interest, etc., they are not subject to interference from public power or others. Medical behavior directly affects the patient’s body, and the patient’s subjective status is not reduced due to the decline in physiological functions. As a human being, he has the right to decide the intervention and limits of medical intervention in his body and other personality areas according to his own will.

During World War II, German Nazi doctors conducted inhumane human experiments on prisoners under the banner of developing medical science. After the war, the International Military Tribunal tried the Nazis’ anti-human medical atrocities. The Nuremberg Code was introduced in this context, which clearly stated that “the voluntary consent of the human subject is absolutely essential” and “during the course of the experiment the human subject should be at liberty to bring the experiment to an end if he has reached the physical or mental state where continuation of the experiment seems to him to be impossible” and so on (14). The atrocities committed by Nazi doctors destroyed the trust between doctors and patients, and the public questioned whether doctors’ actions were all based on the interests of patients. The Nuremberg Code aims to regulate that human experiments must obtain the voluntary consent of the subjects and that the subjects must be informed and explained to ensure that they have full knowledge and understanding of the trial projects and processes. The Nuremberg Code shook the authority of medical paternalism, prompted the medical community to pay more attention to patients’ medical rights, and also shifted the doctor-patient relationship toward the patients. In 1964, the 18th World Medical Association General Assembly adopted the Declaration of Helsinki, which adopted the views of the Nuremberg Code and formally confirmed the right of subjects to self-determination in Article 9 (15). In October 1981, the World Medical Assembly adopted the Lisbon Declaration of Patients’ Rights, which clearly stipulates that patients have the right to make independent decisions about their medical treatment, and formally established and affirmed patients’ autonomous rights and independent subject status in medical activities (16). So far, through the joint efforts of the international community, patients’ independent decision-making on medical measures has generally become the highest principle that must be followed in medical activities.

### 2.2 Policy frameworks on medical autonomy in other countries and regions

With the improvement of the modern legal system, countries and regions around the world have begun to institutionalize patients’ medical autonomy rights. This has since kicked off the legalization of patient autonomy. Patients have begun to be separated from other subjects and enjoy the control of their own medical decisions. Right.

In 1990, the U. S. Congress amended Chapter 18 of Medicare and Chapter 19 of Medicaid in the Social Security Act and passed the Patient Self Determination Act to protect patients’ autonomy in medical decision-making (17). After that, the states of the United States made localized regulations on federal laws according to their own cultural characteristics and historical traditions. For example, the state legislatures of Colorado and Maryland passed the Colorado Patient Autonomy Act and the Maryland Health Care Decision Act in 1992 and 1993, respectively, as laws to protect patients’ autonomy in making medical decisions.

Patient autonomy legislation developed slightly later in Europe than in the United States. In 1997, the Council of Europe adopted the Protection of Human Rights and Dignity of the Human Being with regard to the Application of Biology and Medicine: Convention on Human Rights and Biomedicine, Article 9 clearly states: “the previously expressed wishes relating to a medical intervention by a patient who is not, at the time of the intervention, in a state to express his or her wishes shall be taken into account” (18). Which protects patient autonomy by respecting advance directives and the right to choose a medical proxy. Many countries including the United Kingdom, France, the Netherlands, Norway and Spain have signed the convention. Although different countries have different legislations based on differences in their internal legal systems, most countries’ legislations follow the spirit of this convention. For example, The United Kingdom enacted the Mental Capacity Act in 2005, which stipulates that anyone over 18 years old and capability can choose a medical agent or make advance medical directives when they are conscious; there is also a court of protection to deal with disputes in cases of intervene referee (19). Germany amended Civil Code in 2009, integrating relevant concepts into the Civil Code, further establishing the protection of patients’ autonomy through advance directives and medical caregivers (20).

Asian countries and regions enacted legislation on medical autonomy later than Europe and the United States. The idea of medical autonomy has evolved from the Western world to Eastern society. Asian countries and regions have also gradually realized the importance of patients’ independent medical decisions, and relevant judicial and legislative measures have promoted each other. In the 1992 “Jehovah’s Witnesses Refusal of Blood Transfusion Case” in Japan, the Supreme Court made a final judgment in 2000, the judgment pointed out that when a patient believes that blood transfusion violates his religious beliefs and explicitly refuses medical treatment involving blood transfusion, his right to make such a decision falls within the scope of personality rights and should be respected. Since the doctor failed to fulfill his duty of explanation, he deprived the patient of the right to decide whether to undergo surgery, thereby infringing his personality rights (21). In 2016, South Korea passed the Act on Hospice and Palliative Care and Decisions on Life-Sustaining Treatment for Patients at the End of Life (22), which stipulates the patient’s right to medical autonomy at the end of life, and makes detailed rule designs in terms of applicable subjects, procedures, etc. In 2000, China’s Taiwan region passed the Hospice Palliative Care Act (23) to regulate the medical autonomy of terminally ill patients, and in 2016, it enacted the Patient Right to Autonomy Act (24), which made further provisions on medical consultation, agents, procedures, and other issues regarding terminally ill patients’ refusal of medical life-sustaining measures, artificial nutrition, and fluid feeding, to protect patients’ medical autonomy and the right to a good end.

In summary, we can find that medical autonomy is a product of the development of social civilization and the protection of human rights. Medical measures involve the patient’s life, body, health, privacy and other elements that are most closely related to people. These elements are inseparable from people’s future development, self-improvement and self-creation. In order to ensure the autonomy of patients in medical affairs, many countries and regions around the world have adopted a policy framework to protect patients’ autonomy, ensure that patients decide on medical activities according to their own wishes, and improve the level of human rights protection for patients. These policies respect the patient’s decisions and choices, ensure that patients can decide their own lives according to their own will, and improve the quality of life of patients. It is necessary for China’s legislature to learn from useful experiences in light of national conditions.

## 3 China’s policy framework

China’s legislative activities on patient autonomy started late. It was not until the mid-1990s that Chinese managers and legislators realized the need to protect patient autonomy and gradually formulated a series of relevant legal systems.

The “Hospital Work System” promulgated by the Ministry of Health of China in 1982 stipulates in the “Several Rules for Performing Surgeries”: “Before performing an operation, the patient’s family or unit must sign and agree (surface surgery does not require a signature). When there is no time to obtain the consent of the family or the unit for an emergency operation, the attending physician may sign and the department director or the dean or the deputy dean may approve the operation” (25). From this regulation, we can see that the patient’s family and unit are the main decision-makers for medical treatment, and the patient himself cannot decide his own medical activities, which seriously ignores the subjectivity of the patient. The strangest thing is that the unit is the decision-maker for the patient’s medical activities, which is incredible.

Article 33 of the Regulations on the Administration of Medical Institutions promulgated by the State Council in 1994 stipulates: “When a medical institution performs surgery, special examinations or special treatments, it must obtain the patient’s consent and obtain the consent and signature of his or her family or relatives; when it is impossible to obtain the patient’s opinion, the consent and signature of the family or relatives shall be obtained; when it is impossible to obtain the patient’s opinion and there are no family or relatives present, or when encountering other special circumstances, the attending physician shall propose a medical treatment plan and implement it after obtaining the approval of the head of the medical institution or the authorized person in charge” (26). There has been some progress in this article, which has eliminated the requirement for units to serve as medical decision-makers. However, this article stipulates that in addition to the consent of the patient himself, the medical institution also requires the consent of the family or related persons when carrying out relevant medical activities. This double insurance mechanism is not conducive to the exercise of patient autonomy.

Article 62 of the Implementation Rules of the Medical Institution Management Regulations, issued in August 1994, stipulates: “Medical institutions shall respect the patient’s right to know about their condition, diagnosis and treatment. When performing surgery, special examinations and special treatments, necessary explanations shall be given to the patient. If it is not appropriate to explain the situation to the patient due to the implementation of protective medical measures, the patient’s family shall be notified of the relevant situation” (27). The Implementation Rules stipulates the medical institution’s obligation to provide explanations and the patient’s right to know, which is of great significance to the protection of patient autonomy. However, it does not stipulate the medical institution’s obligation to obtain the patient’s consent and the patient’s right to choose.

In 1998, Article 26 of the Law on Practicing Doctors of the People’s Republic of China stipulates: “Physicians shall truthfully explain the condition of patients or their families, but shall be careful to avoid adverse consequences for patients. Physicians shall obtain approval from the hospital and consent from the patient or his family before conducting experimental clinical treatment” (28). This is the first time that Chinese law has formally stipulated doctors’ obligation to inform and explain, and established the rules of informed consent.

Article 20 of the Medical Cosmetic Service Management Measures issued by the Ministry of Health in 2001 stipulates: “Before a practicing physician provides treatment to a patient, he or she must inform the patient or his or her relatives in writing of the indications, contraindications, medical risks and precautions of the treatment, and obtain the signed consent of the patient or guardian. Medical cosmetic projects shall not be implemented for persons with no or limited capacity without the consent of the guardian” (29). This is the first regulation that stipulates patient autonomy in the special medical field, and is of great significance to patient protection.

Article 11 of the Regulations on Handling Medical Accidents promulgated by the State Council in 2002 stipulates: “In medical activities, medical institutions and their medical personnel shall truthfully inform patients of their conditions, medical measures, medical risks, etc., and promptly answer their inquiries; however, adverse consequences for patients should be avoided” (30). This article extends the obligation to inform and explain from clinical medicine to the entire medical activities, requiring medical institutions and their medical staff to fulfill their obligation to explain in detail.

Article 55 of the Tort Liability Law of the People’s Republic of China, promulgated in 2009, stipulates: “Medical personnel shall explain the condition and medical measures to the patient during diagnosis and treatment. If surgery, special examinations or special treatments are required, medical personnel shall promptly explain the medical risks, alternative medical plans, etc. to the patient and obtain their written consent; if it is not appropriate to explain to the patient, the patient’s close relatives shall be explained and their written consent shall be obtained” (31). Compared with the previous Medical Practitioner Law and Regulations on Medical Accident Handling, the main change is that “close relatives” have replaced “relatives.”

The Basic Standards for Medical Record Writing issued by the Ministry of Health in 2010 made detailed provisions for the signing of consent forms. Article 10 stipulates: “For medical activities that require the patient’s written consent, the patient himself shall sign the informed consent form. If the patient does not have full civil capacity, his legal representative shall sign; if the patient is unable to sign due to illness, the person authorized by him shall sign; in order to save the patient, if the legal representative or authorized person is unable to sign in time, the person in charge of the medical institution or the authorized person may sign”. (32) This article links the patient’s capacity to make independent medical decisions with civil capacity.

Article 1,219 of the 2020 Civil Code of the People’s Republic of China stipulates: “Medical personnel should explain the condition and medical measures to the patient during diagnosis and treatment. If surgery, special examinations, or special treatments are required, medical personnel should promptly explain the medical risks, alternative medical plans, etc. to the patient and obtain their explicit consent; if it is impossible or inappropriate to explain to the patient, the patient’s close relatives should be explained and their explicit consent should be obtained”. (33) Compared with the Tort Liability Law, the Civil Code simplifies the formal conditions for patient consent and adds the provision that “it is not appropriate to explain to the patient”. This protective medical care is more conducive to protecting the physical and mental health of patients and avoiding excessive psychological pressure on patients.

From the changes in the above policy framework, we can find that after a long period of development, China’s medical decision-making has gradually become modernized and legalized. These policies promote the medical decision-making model to keep approaching the standards of Western medical civilization. However, we must admit that China and the West have differences in medical decision-making models, and different cultural traditions have led to these differences. The patient autonomy model influenced by Western liberal thought emphasizes the patient’s independence, separates the intimate relationship between the patient and his family, and ignores the family’s involvement in injuries and illnesses.

## 4 Familism and China’s medical decision-making model

Due to the invasive nature and risks of medical measures, in addition to improving the patient’s physiological functions, they may also damage the patient’s life, health, body, privacy, etc. Generally speaking, medical decisions are made by the patients themselves, but patient autonomy is a seemingly beautiful institutional design. In medical practice, many patients are not capable of making medical decisions, such as minors, patients with severe mental disorders, patients with severe intellectual disabilities, and people in a coma. Their medical decisions must be made by others. In Chinese society, the influence of “family culture” is deeply rooted. In most cases, medical decision-making adopts the medical familism model, which protects patients to a certain extent and avoids the dilemma of patients facing the disease independently. The model of families making medical decisions for their patients is already a long-standing one in medical history. In ancient Rome, in addition to guardianship, the father was also responsible for treating the illnesses of family members and served as the doctor of family members. Cato Maior claimed to have a medical notebook that he used to heal his son, slaves, and family members (34). Similar medical familism is also common in some Asian countries and regions influenced by Confucianism (35). However, it must be acknowledged that medical familism sometimes oppresses patients’ autonomy.

### 4.1 Familism and medical practice

For thousands of years, under the background of small peasant economy, Chinese people have completely relied on “family” to live, and have adhered to the family-oriented life belief of “living for family and existing at home” (36). In Chinese society, patients are not always isolated and helpless due to their illness. Their families often provide help to them and play an important role in medical activities. Under the family-oriented style, the birth, aging, illness and death of family members are extremely important matters for the family, and they substantially affect the future development of the family. If a family member falls into misfortune due to injury or illness, the extended family members will give patients care and help patients overcome the difficulties. Injury and illness are not the suffering of the patient alone, but a challenge that the family must face together (37). In addition to providing psychological support for the patient, the family often has to bear the patient’s medical expenses. For ordinary families, the total economic income of the family is limited, and patients are not always able to bear the high medical expenses alone. For example, minors, the older adult and the mentally handicapped, etc., these vulnerable groups often need the family finances to bear part or even all of the medical expenses. In the real society, families with financial constraints cannot even afford the high medical expenses with the whole family’s efforts, and tragedies of poverty due to illness often occur. For malignant diseases, even if the patient receives treatment, complete cure is not guaranteed, and patients often die after treatment. The medical expenses of patients divide the family’s overall disposable income, affecting the subsequent operation of family life. Once the issue of property expenditure is involved, it is very easy to cause family discord and relationship breakdown. Not only that, the occurrence of the disease weakens the patient’s psychological state, and the medical dilemma triggers the patient’s guilt. When he becomes a burden to the family, it is better to give up treatment and save the family as a whole, rather than bear the risk of losing both money and money after receiving treatment. What’s more, patients choose to commit suicide to avoid dragging down their families.

In Chinese society, the family occupies an important position in human life, which demonstrates the fullness and harmony of the Confucian family concept. Confucianism advocates the overall care and wisdom of the family on major life issues such as patient medical care, child rearing, and marriage. The family is the best authority for making these decisions, and this authoritative status of the family reflects the moral and ontological priority of the family (38). As a member of the family, the development of the patient’s illness is related to the subsequent development of the daily life of the entire family (39). The patient’s medical activities are not just his or her own business, but a public affair within the family. The patient does not have full decision-making right over his or her own illness, and the development of his or her medical activities is decided collectively by family members (40).

### 4.2 Familism shapes China’s medical decision-making model

According to the provisions of Article 1,219 of the Civil Code, when special medical activities are carried out, if the patient lacks awareness or it is inappropriate to inform psychologically fragile patients based on humanistic care, the obligation to inform the patient’s close relatives shall be fulfilled and authorization from the close relatives shall be obtained. The subject restriction of medical decision-making agents stipulated in this article reflects the concept of familism. This family relationship based on blood relationship is more reliable than other social relationships. Family members (especially close relatives) are more likely to make medical decisions that are in the best interests of patients than other subjects. As the patient’s advocate, the medical decision agent cannot make any claims without the patient’s prior consent. Instead, he or she needs to negotiate with family members (including the patient himself in certain circumstances) to make medical decisions together. In the “family concept” shaped under the influence of Confucianism, family members jointly build a social entity, family members care and look after each other, and the collective wisdom of the family can often contribute to choices that are in the best interests of the patient.

As an important carrier of a person’s life journey, family has important life value for individuals. Family members support and depend on each other and jointly promote the progress and development of the family. This group of individuals gathered together based on blood relations have a higher degree of altruism. Mutual help among relatives is out of ethical obligations and based on the emotion of kinship (41). Biologist Hamilton’s kin selection theory holds that altruistic behavior generally occurs between relatives with blood ties, the closer the blood relationship, the stronger the altruistic tendency between each other (42). When a patient suffers from a disease, his body and mind are attacked by the disease, and his mental endurance is greatly reduced. If he is immediately informed of his bad condition (especially serious injuries), it may increase the patient’s physical and mental burden and easily cause the patient lost his belief in survival and refused to cooperate with the doctor and family in receiving follow-up medical treatment. For example, when a patient suffers from a malignant tumor, those who are unaware of the severity of their condition are more likely to maintain a more optimistic attitude and cooperate with treatment, which is beneficial to treatment to a certain extent. Once informed, the fear and worry about the malignant disease will be detrimental to the development of the patient’s injury and illness, and may even lead to extreme suicidal behavior. In major medical activities involving the life and health of patients, it is often the patients’ families who negotiate with doctors on their behalf. After the doctors reach an agreement with the patients’ families, they conceal the information from the patients, blocking the actual medical information from flowing to the patients, preventing the patients from immediately knowing about their serious conditions, and waiting for the right time to inform them indirectly and euphemistically, or even concealing it completely. Such practices may seem to deprive patients of their autonomy, but they are quite common in medical practice and have inherent rationality in Chinese society.

## 5 Suggestions

In the long and short life course of human beings, “birth, aging, illness and death” are inevitable events. The core of personality lies in self-determination. As a person, being able to decide his own medical activities according to his own will is of great significance to his personality shaping and development. As Isaiah Berlin said: “I wish my life and decisions to depend on myself, not on external forces of whatever kind. I wish to be the instrument of my own, not of other men’s, acts of will. I wish to be a subject, not an object; to be moved by reasons, by conscious purposes, which are my own, not by causes which affect me, as it were, from outside” (43). Due to the institutional defects in China’s policy framework, there are still obstacles to the realization of patients’ medical autonomy. The legal protection of vulnerable groups can better demonstrate the value pursuit of equality before the law. This article puts forward the following suggestions from the institutional level in order to enhance the autonomy of Chinese patients in medical decision-making.

### 5.1 Reconstruct the criteria for determining medical decision-making capacity

In the medical field, whether patients can make independent decisions on medical activities requires corresponding capabilities, namely medical decision-making capacity. Medical decision-making capacity is a necessary condition for patients to make independent decisions on medical activities. Under Chinese law, Article 10 of the Basic Standards for Medical Record Writing unreasonably links medical decision-making capacity with civil capacity. Article 17 of the Civil Code stipulates that natural persons over the age of 18 are adults; Article 18 stipulates that adults are persons with civil capacity and can independently perform civil legal acts; Article 21 stipulates that adults who cannot recognize their own actions are persons with no civil capacity; Article 22 stipulates that adults who cannot fully recognize their own actions are persons with limited civil capacity. From the framework of China’s Civil Code, we can find that the Chinese legislature regards full civil capacity as a necessary condition for the capacity to make medical decisions. Only people with full capacity for civil conduct have the capacity to make medical decisions, while people with incomplete capacity for civil conduct do not have the capacity to make medical decisions. There are obvious legal loopholes in such a provision. The age span of minors is large, and it is too hasty to classify minors as persons without the capacity to make medical decisions without taking into account their individuality. As minors grow older, they gradually move closer to adults, and their cognitive and rational capacity tend to be more perfect. Especially for older minors, their thinking level is basically the same as that of adults. The same applies to adults with mild cognitive impairment. Even if their rational abilities are insufficient, there are still matters that they can make rational judgments about. China’s regulations ignore patients’ actual cognitive levels and ignore their actual needs to normalize their social lives. In addition, in daily life, people with full civil capacity may fall into a coma or lose consciousness due to injuries, illness, alcoholism, etc. At this time, they have no capacity to make medical decisions, which leads to a logical paradox.

In comparative law, British and American court cases have recognized that minors and adults with cognitive disabilities have partial medical decision-making capabilities. In the British case “Gillick v. West Norfolk and Wisbech Area Health Authority,” the judge established the “Gillick Capacity Rule”. The British House of Lords believed that parental supervision over their minor children gradually fades as their children grow up. When children have the recognition and intelligence to fully understand the nature and consequences of medical behavior, parental supervision over their children’s medical decisions ends (44). The judgment in the Gillick case affirmed the medical decision-making capacity of minors under the age of 16. If the minor meets the requirements of having sufficient cognition and understanding of the proposed medical behavior, he or she will be deemed to have the medical decision-making capacity to decide on the medical behavior, and can independently decide on medical matters without the consent of the legal representative. In the American case of “Bakker v. Welsh,” the Michigan Supreme Court held that although parents are the legal guardians of their minor children, given that the 17-year-old patient in this case was mature enough to decide on his own for surgical procedures, the father’s consent to the surgery was unnecessary. Therefore, the hospital’s performance of surgery on the minor patient without the father’s consent was not illegal (45).

In the British “Re C (Adult: Refusal of Medical Care)” case, the court held that although the perpetrator was a mentally disordered person and his cognitive capability was impaired due to schizophrenia, his remaining capability was sufficient to understand the nature and consequences of amputation surgery, etc. matter, so his medical decision to refuse amputation is valid (46). The Convention on the Rights of Persons with Disabilities takes respect for inherent dignity and individual autonomy as its primary principles and requires States Parties to recognize that persons with disabilities enjoy legal capacity on an equal basis with others in all aspects of life in order to promote respect for the inherent dignity of persons with disabilities (47). Respecting the autonomy of adult people with cognitive disabilities should ensure that they can act according to their own will within the scope of their abilities, shape and develop their own personalities, and return to normalization of social life.

In summary, this paper believes that medical decision-making capacity should be based on the patient’s actual cognitive and judgment abilities. As long as the patient has the corresponding capacity to identify the proposed medical behavior, can understand the nature, risks, consequences and other matters of the medical behavior, analyze the pros and cons based on this, make rational judgments, and can communicate to the outside world, he or she has the corresponding medical decision-making capacity. In this sense, Article 10, Paragraph 1 of the Basic Standards for Medical Record Writing should be amended as follows: “For medical activities that require the patient’s written consent, if the patient has the capacity to recognize such medical activities, the patient himself shall sign the informed consent form; when the patient does not have the capacity to recognize such medical activities, his legal representative shall sign; when the patient is unable to sign due to illness, the person authorized by him shall sign; in order to rescue the patient, if the legal representative or authorized person is unable to sign in time, the person in charge of the medical institution or the authorized person may sign.”

### 5.2 Establish an assessment mechanism for medical decision-making capacity

The level of medical decision-making capacity is based on factors such as the patient’s intelligence, mental health status, disease, and treatment content. These factors have different intensities of influence on medical decision-making capacity, and together they form the basis for judging medical decision-making capacity. In medical practice, whether a patient has the decision-making capacity for the proposed medical measures requires a case-by-case review by the doctor based on the specific circumstances. The generally recognized review elements for medical decision-making capacity include: (A) understanding (whether the patient understands the information related to the proposed medical measures); (B) identification (whether the patient understands and identifies his or her own clinical condition related to the treatment plan); (C) reasoning (whether the patient can make rational judgments and decisions about the proposed medical measures); (D) expression of choice (whether the patient can express the reasons for making the decision and whether he or she can ask and answer relevant questions) (48). In addition to relying on the doctor’s experience and judgment, the assessment of medical decision-making capacity is often made with the help of relevant capacity assessment tools, such as the MacArthur Capacity Assessment Tool for Treatment (MacCAT-T), the Capacity to Consent to Treatment Instrument (CCTI), the Hopemont Capacity Assessment Interview (HCAI), the Montreal Cognitive Assessment (MoCA), etc. (49). It is difficult to determine a patient’s medical decision-making capacity in a flexible system influenced by multiple factors. It is necessary for China to establish an assessment mechanism for medical decision-making capacity, determine the patient’s level of medical decision-making capacity for specific diagnosis and treatment content, and create conditions for patients to make independent medical decisions.

### 5.3 Implement advance medical directive system

Medical intentions carry the patient’s expectations for the future, concern whether the patient agrees that medical activities will intervene in his or her personality and the limits thereof, and reflect the patient’s autonomous demands. Advance medical directives can extend patients’ autonomy after they lose the capacity to make medical decisions, thereby helping to achieve their best interests. Chinese social organizations have been promoting and advocating advance medical directives. In 2006, a group of public welfare individuals established the “Choice and Dignity” public welfare website, and on June 25, 2013, the Beijing Living Advance Directive Promotion was established on the basis of this website. Association, which calls on the public to fill in the text “My Five Wishes” to make a “living will” so that individuals can decide independently whether to use a ventilator or other medical life-sustaining measures at the end of life (50). However, since Chinese law does not stipulate an advance medical directive system, the effectiveness of advance medical directives has not been recognized by law. Even if a patient has made a complete and detailed advance medical directive and made adequate arrangements for medical measures after he or she loses the capacity to make medical decisions, the hospital and the patient’s immediate family members may not be aware of the existence of the advance medical directive. Even if they are aware of it, the lack of legal effect poses a hidden danger for the hospital or the patient’s immediate family members to violate the advance medical directive made by the patient.

This situation took a turn for the better in 2022, when Shenzhen revised the Regulations on Medical Treatment in the Shenzhen Special Economic Zone, Article 78 of which stipulates: “When a medical institution receives a living will from a patient or his/her immediate family, it shall respect the patient’s wishes when implementing medical measures at the end of the patient’s incurable illness or at the end of his/her life” (51). This provision provides a legal basis for the implementation of the advance medical directive system. Other cities in China should follow Shenzhen’s approach and promote it nationwide, and may gradually improve it to protect patients’ autonomy. Therefore, China’s legislature should actively promote the legalization of the advance medical directive system, formulate national unified standards for advance medical directives, and make clear provisions on necessary matters such as applicable subjects, scope of application, applicable procedures, and relief measures.

### 5.4 Grant medical institutions the authority to implement special medical interventions

In medical practice, not all medical decisions are based on the true wishes of the patients. There are also cases where patients make wrong and unfavorable medical decisions due to external coercion or deception. For example, in order to inherit their property as quickly as possible, the patient’s relatives may mislead the patient to give up treatment and end their life as soon as possible, thus causing the patient to make irrational medical decisions. In the above-mentioned “Nantong Welfare Institution’s Removal of a Young Girl’s Uterus” case, the welfare institution’s leaders chose to remove the girl’s uterus simply to reduce the difficulty of care, thereby infringing upon the girl’s body. The means implemented by the welfare institution and the legal interests protected were seriously inconsistent with the principle of proportionality. If the doctor had been able to intervene, this tragedy might have been avoided. Therefore, how to protect patients’ medical rights after making inappropriate medical decisions is an important issue that the legislature needs to consider.

Doctors use benevolent techniques to save lives. Medical activities have a highly ethical nature, and medical institutions cannot blindly comply with medical decisions without reviewing them. Some patients with cognitive impairment lack rational capacity and are easily induced and deceived by others, leading to irrational medical decisions. Medical decision agents may also make medical decisions that go against the patient’s wishes and harm the patient’s rights. In order to protect patients’ autonomy and promote the harmonious development of doctor-patient relationship, China’s legislature should grant medical institutions the authority to implement special interventions, so that medical institutions can make reasonable interventions when false medical decisions and improper agency decisions harm the rights and interests of patients.

## 6 Conclusion

With the evolution of social civilization, concepts such as personal dignity and personal autonomy have penetrated into people’s minds, medical paternalism has gradually faded, medical rights have gradually shifted to the patients themselves, and patient autonomy has become a legal norm and a consensus in the medical affairs community. In Chinese society, Confucianism has constructed a family-oriented ideology. In the medical familism model, medical decision-making integrates patient autonomy and the overall will of the family, emphasizing the contribution of collective wisdom. Although the medical familism model that is prevalent in Chinese society has its inherent rationality, the intensity of family wisdom intervention should be limited and kept within a reasonable range. Although the overall wisdom of the family is in line with the best interests of the patient as much as possible, this is only a seemingly beautiful rule setting. The overall wisdom of the family is essentially still “other-oriented,” which is different from the “autonomy” of the patient. Driven by interests, it is inevitable that there will be risks of harming the interests of the patient. China should promote the transition from “strong familism style” to “weak familism style” from the policy framework, and transform family participation from an “intervention role” to an “assistance role”. By improving policies and measures, exploring the balance between family intervention and patient autonomy, enhancing patient autonomy in medical decision-making.

## Data Availability

The original contributions presented in the study are included in the article/supplementary material, further inquiries can be directed to the corresponding author.
